# Gentle, fast and effective crystal soaking by acoustic dispensing

**DOI:** 10.1107/S205979831700331X

**Published:** 2017-03-06

**Authors:** Patrick M. Collins, Jia Tsing Ng, Romain Talon, Karolina Nekrosiute, Tobias Krojer, Alice Douangamath, Jose Brandao-Neto, Nathan Wright, Nicholas M. Pearce, Frank von Delft

**Affiliations:** a Diamond Light Source, Harwell Science and Innovation Campus, Didcot OX11 0DE, England; b Structural Genomics Consortium (SGC), University of Oxford, Oxford OX3 7DQ, England; c Department of Biochemistry, University of Johannesburg, Aukland Park, Johannesburg 2006, South Africa

**Keywords:** fragment screening, crystal soaking, acoustic droplet ejection, Diamond Light Source I04-1, Structural Genomics Consortium, XChem

## Abstract

The steady expansion in the capacity of modern beamlines for high-throughput data collection, enabled by increasing X-ray brightness, capacity of robotics and detector speeds, has pushed the bottleneck upstream towards sample preparation. Even in ligand-binding studies using crystal soaking, the experiment best able to exploit beamline capacity, a primary limitation is the need for gentle and nontrivial soaking regimens such as stepwise concentration increases, even for robust and well characterized crystals. Here, the use of acoustic droplet ejection for the soaking of protein crystals with small molecules is described, and it is shown that it is both gentle on crystals and allows very high throughput, with 1000 unique soaks easily performed in under 10 min. In addition to having very low compound consumption (tens of nanolitres per sample), the positional precision of acoustic droplet ejection enables the targeted placement of the compound/solvent away from crystals and towards drop edges, allowing gradual diffusion of solvent across the drop. This ensures both an improvement in the reproducibility of X-ray diffraction and increased solvent tolerance of the crystals, thus enabling higher effective compound-soaking concentrations. The technique is detailed here with examples from the protein target JMJD2D, a histone lysine demethylase with roles in cancer and the focus of active structure-based drug-design efforts.

## Abstract 2

A high-throughput method is described for crystal soaking using acoustic droplet ejection, and its effectiveness is demonstrated.

## 1. Introduction   

Obtaining protein–ligand complexes, the workhorse experiment in structure-based ligand design (SBLD), relies on two methods for achieving the prerequisite crystals: crystal soaking and co-crystallization (Hassell *et al.*, 2007 ▸). Co-crystallization is achieved by adding the small molecule of interest to the protein prior to setting up a crystallization experiment, or by simply including it as a component in the crystallization condition. The potential ligand is free to bind to the protein in solution prior to the formation of a crystal lattice, allowing the freedom for potential structural changes. Crystal soaking is the process of taking pre-grown crystals and soaking them with the small molecule of interest. The potential ligand can access the binding sites by diffusing through solvent channels within the crystal lattice, as long as the sites are not involved in crystal packing or otherwise obscured (Danley, 2006 ▸).

A co-crystallization method for preparing large numbers of crystals for screening by X-ray crystallography is the use of ‘dry’ co-crystallization, in which small-molecule libraries are dispensed to the sitting-drop locations of a crystallization plate before removing the solvent by evaporation (Gelin *et al.*, 2015 ▸). This leaves compound-coated surfaces ready for solubilization by the crystallization droplet, providing the potential for co-crystallization with the compound. X-ray diffraction data can then be collected from crystals *in situ* in the crystallization plate or after retrieving them into cryoloops.

Crystal soaking tends to be experimentally simpler, since it only requires crystals to be available, and also often facilitates the scaling up of the number of small molecules analysed, since it is frequently possible to generate large numbers of crystals of the same form from a known crystallization condition (Hassell *et al.*, 2007 ▸). A common obstacle is the low solubility of many compounds in aqueous solutions, requiring organic solvents such as dimethylsulfoxide (DMSO) for solubilization (Danley, 2006 ▸), which however alter the chemistry of the crystal drop and tend to affect the integrity of the crystal. Thus, the basic challenge of crystal soaking is how to introduce the compound to the crystal without destroying the crystal. One reported approach has been to use a laser to open a small aperture in the thin-film support behind a crystallization droplet (Zander *et al.*, 2016 ▸). The application of a volume of solvent (containing compound) to the rear side of the film allows the solvent to slowly diffuse through the aperture, with the slow equilibration times providing higher tolerated solvent concentrations of the crystals.

A technique that has been gaining utility for protein crystal applications is acoustic droplet ejection, a liquid-handling approach that relies on ultrasound pulses focused towards the surface of a liquid, thereby ejecting nanolitre or smaller volume droplets (Ellson *et al.*, 2003 ▸). The precision and volume scales of acoustic transfer have enabled new develop­ments in protein crystallography, including performing small-volume crystallization experiments in crystallization plates (Wu *et al.*, 2016 ▸; Villaseñor *et al.*, 2012 ▸) or directly on data-collection mounts (Yin *et al.*, 2014 ▸) and transferring pre-formed crystals into mounts (Cuttitta *et al.*, 2015 ▸) or directly into the very short pulse of an XFEL beam (Roessler *et al.*, 2016 ▸). Acoustic droplet ejection has also been used to prepare high-density crystallizations for *in situ* fragment screening (Teplitsky *et al.*, 2015 ▸) where the precision and small volume handling of acoustic droplet ejection were used to fit 1728 crystallization experiments into a single microplate, and the same number of unique compounds could be added to existing crystals for soaking experiments, or added prior to crystallization and before drying as described above for dry co-crystallization. *In situ* data collection coupled with automatic alignment of the crystal plate to position crystallization droplets into the X-ray beam has the potential to yield very high throughput X-ray fragment-screening experiments.

An extreme application of crystal soaking is in crystal-based fragment screening. Fragment methods involve the screening of a protein target against a library of small molecules typically under 300 Da in size (Congreve *et al.*, 2003 ▸). Its advantage is that the probability of binding is increased owing to the smaller, less complex nature of the molecules (Patel *et al.*, 2014 ▸), with chemical elaboration being performed on hits to improve potency (Erlanson *et al.*, 2016 ▸). Its disadvantage is that because of the weak binding nature of the fragments, a screening method with high sensitivity is required for detection. X-ray crystallography is unrivalled for sensitivity, revealing the binding of weakly interacting molecules where other techniques fail (Erlanson *et al.*, 2016 ▸). In this regard it is the ideal method for fragment screening given how the traditional logistical overheads for large-scale X-ray experiments are falling away with the widespread availability of fast pixel-array detectors and high-capacity robotic sample changers (Patel *et al.*, 2014 ▸).

We investigated the utility of acoustic droplet ejection for providing a gentle method for crystal soaking at high solvent concentrations, with the requirement of also being rapid and robust for routine large-scale crystal soaking as part of the XChem fragment-screening user facility at Diamond Light Source.

## 2. Methods   

### 2.1. Overview of approach   

Our technique for soaking by acoustic dispensing entails transferring compounds in solvent to crystals in sitting-drop plates using an Echo 550 (Labcyte, Inc., Sunnyvale, Califrnia, USA). Subsequent steps proceed as usual, with drops allowed to soak for a period of time before crystals are harvested and cryocooled and X-ray diffraction data are collected.

**Figure 1:**
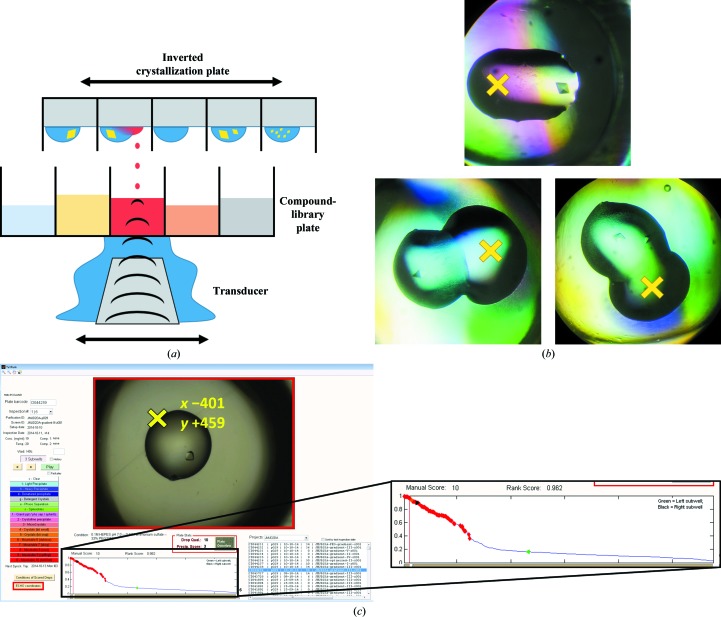
Acoustic droplet ejection allows precise and selective targeting of crystallization droplets. (*a*) Schematic representation of acoustic droplet ejection for crystal soaking using the Labcyte Echo. (*b*) Crystallization droplets (200 nl initial volume) containing protein crystals with acoustically added solvents. Initially, 135 nl of 100 m*M* compound in DMSO was dispensed with the offset targeting approach to the indicated location (yellow ‘X’). Later, 50 nl of cryoprotecting solvent (ethylene glycol) was added at the same location. (*c*) The *TeXRank* interface showing a crystallization drop containing a single JMJD2D crystal. Clicking a location records the acoustic dispensing target for both compound-containing solvents and cryoprotectants (DMSO and ethylene glycol, respectively, in this study). The yellow ‘X’ and *xy* coordinates have been added for clarity. The expanded section shows the ranked plot of crystal images.

The Echo operates by moving a transducer below the stationary compound-library plate (source plate) and focusing sound pulses at the meniscus of the solution in the requested well, resulting in solvent droplets being ejected upwards (red dots in Fig. 1 ▸ *a*). The fixed-frequency sound pulse from the transducer in the Echo 550 produces a fixed-sized 2.5 nl droplet, and larger transfer volumes are achieved by dispensing multiple drops of 2.5 nl at a rate of 200 Hz. The inverted sitting-drop crystallization plate (destination plate) is moved above the compound-library plate to position the requested target position above the stream of solvent droplets; the relevant wells need to be uncovered during this process.

In this application, the positional precision of the Echo is relied on to target solvent away from potentially sensitive protein crystals and towards the drop edges (Fig. 1 ▸ *b*). The compounds used here are dissolved in DMSO at 100 m*M* concentration and placed in Labcyte 1536-well source plates. Cryoprotection, when required, is also performed with the Echo by acoustic transfer (to the same targeting locations) directly from a 100% solution of ethylene glycol in a 384-well source plate using a mode compatible with viscous solvents (glycerol percentage mode).

### 2.2. Dispense volumes and tolerated concentrations   

Although the Echo has high precision (<8% CV) for small-volume transfers (Ellson *et al.*, 2005 ▸), an accurate estimate of the final concentration of solvent or compound after acoustic transfer requires the volume of the crystallization drop to be known. Instead, only the initial drop volume prior to vapour diffusion is available. In a vapour-diffusion experiment, the drop volume of a 1:1 protein:reservoir solution will typically reduce to approximately half the original volume, although the exact end volume depends on the presence of solutes in the protein component, which modify the equilibrium point (Luft & DeTitta, 2008 ▸). This is especially true when PEG solutions are used in the reservoir, since PEGs do not reduce the vapour pressure of water as effectively compared with salts. Establishing such details for many different crystallization systems is not practicable, and therefore no attempt was made here to estimate final drop volumes.

Instead, we perform a solvent-tolerance screen on new conditions to determine the exact amounts of solvent tolerated by the crystal system under the acoustic dispensing conditions (discussed further in §[Sec sec3.6]3.6). It should nevertheless be highlighted that the solvent percentages reported throughout this text are the final percentage concentration (*v*/*v*), calculated based on the initial drop volume, and are likely to be underestimates of the true final concentration by up to half in the case of 1:1 protein:reservoir drops.

### 2.3. Details of acoustic targeting   

The Labcyte *Plate Reformat* software allows *xy* offset values to be specified, in micrometres, that can be used to specify a target location for acoustic dispensing away from the default centre of the well. In order to build a list of targeted locations, the crystallization plates were imaged during incubation (Rigaku Minstrel) and the images were analysed with *TeXRank* (Ng *et al.*, 2014 ▸). *TeXRank* uses texture-analysis and machine-learning methods to rank drops by likelihood of containing a crystal, which greatly facilitates drop selection by ranking the most interesting drops at the beginning of the inspection list (expanded section in Fig. 1 ▸ *c*) presented by the *TeXRank* visualization interface. Additionally, *TeXRank* identifies the centre of each drop-containing lens well from the image, which provides an origin for setting the precise physical location relative to the centre of the well to be targeted by the Echo dispensing. The pixel-to-micrometre scale is also calibrated for a given plate imager.

The *TeXRank* interface was modified to support targeting by a single mouse click. Clicking on a specific point of the image (the yellow ‘X’ in Fig. 1 ▸ *c*) (i) registers the drop for inclusion in the experiment (contains a suitable crystal), (ii) records the target location for acoustic dispensing, an *xy* coordinate offset from the origin (well centre) in micrometres, and (iii) brings up the next well in the ranked list. Thus, a crystallographer can very rapidly (in minutes) build up the whole list of very precise dispensing target locations. This list, consisting of plate name (usually a barcode), well location and *xy* offset coordinates, is exported from *TeXRank*. The file format output by *TeXRank* is detailed in Supporting Information §S1.

### 2.4. Configuring the Echo   

The sitting-drop crystallization plates used were SWISSCI 3-drop plates (Ng *et al.*, 2016 ▸). The Echo is compatible with arbitrary destination plates which can be configured within the software by the creation of a labware definition for the plate. The SWISSCI 3-drop plates (Supplementary Fig. S1) have 96 positions with four subwells (three crystallization wells and one reservoir well) per position. The labware definition for such a plate can be created and used within the Labcyte *Array Maker* software; however, this software is not compatible with the targeting offset values, nor does it have the ability to import transfer lists created outside the software. Instead, the plate was defined in a 384-well format (red numbering in Supplementary Fig. S1) and used with the Labcyte *Plate Reformat* software, which does allow the targeting of offset values and the import of transfer lists. To address the technical complication that wells are non-uniformly distributed horizontally, with subwell *d* (Supplementary Fig. S1) positioned 700 µm off-centre, an additional offset correction of 700 µm is applied to these positions (even-numbered columns in 384-well format). The Echo labware definition for the SWISSCI 3-drop plate definition in 384-well format is available for download from the Diamond XChem website (http://www.diamond.ac.uk/Beamlines/Mx/Fragment-Screening.html). Further fine-tuning of accuracy was performed by iterative dispensing and adjustment of the plate definition, and by mechanical calibration of the destination plate carrier. Lists of acoustic dispensing targets from *TeXRank* can be matched to a list of compounds with any spreadsheet tool. The file format required for upload to the Echo is detailed in Supporting Information §S2.

### 2.5. Crystallization, data collection, processing and refinement   

JMJD2D (KDM4D) was expressed and purified as described previously (Bavetsias *et al.*, 2016 ▸), with a protein buffer consisting of 10 m*M* HEPES pH 7.5, 0.5 *M* NaCl, 5% glycerol, 0.5 µ*M* TCEP. Crystals were grown in SWISSCI 3 Lens crystallization sitting-drop plates at 20°C by mixing 50–100 nl of 11 mg ml^−1^ protein solution in a 1:1 ratio with 50–100 nl reservoir solution consisting of 0.1 *M* HEPES pH 7.0, 0.15 *M* ammonium sulfate, 26–37%(*w*/*v*) PEG 3350 and placing the drops over 20 µl reservoir solution. Crystals appeared in 1–3 d. Crystal soaking was performed by acoustic transfer using a Labcyte Echo 550, with the compounds/solvent targeted away from the crystals and towards the drop edges (using *TeXRank* for targeting). Ethylene glycol was added for cryoprotection using the Echo to a final concentration of 20%(*v*/*v*) (calculated from the initial drop volume) and dispensed to the same targeting locations as used for compounds. The JMJD2D crystals diffracted to 1.3–1.6 Å resolution in space group *P*4_3_2_1_2, with typical unit-cell parameters *a* = 71.5, *c* = 150 Å and with one JMJD2D molecule in the asymmetric unit.

X-ray diffraction data were collected on beamline I04-1 at Diamond Light Source and were processed using the Diamond autoprocessing pipeline, which utilizes *xia*2 (Winter, 2010 ▸), *DIALS* (Waterman *et al.*, 2016 ▸), *XDS* (Kabsch, 2010 ▸), *POINTLESS* (Evans, 2006 ▸) and *CCP*4 (Winn *et al.*, 2011 ▸). Electron-density maps were generated using *XChemExplorer* (Krojer *et al.*, 2017 ▸) *via DIMPLE* (Wojdyr *et al.*, 2013 ▸). Ligand restraints were generated with *AceDRG* (Long *et al.*, 2017 ▸) and ligand binding was detected with *PanDDA* (Pearce *et al.*, 2016 ▸), with ligands built into *PanDDA* event maps. Iterative refinement and manual model correction was performed using *REFMAC* (Murshudov *et al.*, 2011 ▸) and *Coot* (Emsley *et al.*, 2010 ▸), respectively.

## 3. Results and discussion   

### 3.1. Acoustic dispensing is effective for soaking   

In order to confirm that acoustic dispensing could be used effectively to obtain protein–ligand complexes, we performed soaking experiments using crystals of the histone demethylase JMJD2D and a known binder reported as part of a structure-based drug-design effort targeting the histone lysine demethylase (KDM) family [compound 30a from Bavetsias *et al.* (2016) ▸, named KDOAM16 here]. A number of acoustic transfers were performed to different crystal drops, with transfer volumes ranging from 5 to 50 nl (directly from a 100 m*M* DMSO stock), and overnight soaks. Electron-density maps from all experiments clearly revealed strong positive difference density for the ligand in the binding site (data not shown). Refinement of the ligand showed that the binding pose and protein–ligand interactions of KDOAM16 were identical to those reported previously (PDB entry 5f5a). This formed the basis of further experiments to identify optimal soaking parameters and experimental procedures, with a view to deploying the technique as a robust and central component of the XChem fragment-screening facility at Diamond Light Source.

### 3.2. Exploiting positional precision to enhance solvent tolerance   

**Figure 2:**
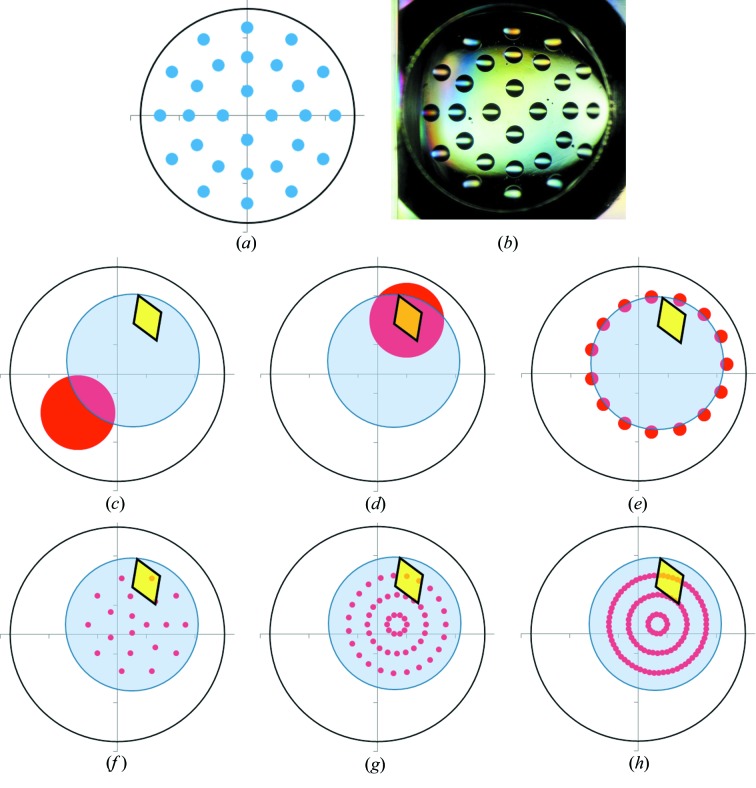
Positional precision of acoustic transfer allows exploration of the influence of complex dispensing patterns on the solvent tolerance of protein crystals. (*a*) The requested pattern of dispensing and (*b*) the resulting 2.5 nl drop pattern within a subwell of a sitting-drop plate. (*c*–*h*) Schematic representations of different dispensing patterns showing a crystallization droplet (blue circle), a protein crystal (yellow diamond) and the solvent target locations (red spots) within a sitting-drop well (outer circle). Dispensing patterns investigated were (*c*) an offset location away from the crystal, (*d*) direct targeting of the crystal, (*e*) a ring pattern around the drop edge (15 target locations) or multiple ring patterns across the drop, increasing in target density and final DMSO concentration, with (*f*) 20% final DMSO concentration (20 target locations), (*g*) 40% (43 target locations) or (*h*) 60% (120 target locations).

The ability of the Echo to dispense solvent to arbitrarily requested locations within a subwell, or to dispense with a complex pattern in 2.5 nl units (Fig. 2 ▸), opens up possibilities for crystal-soaking experiments that are not possible to perform by hand. On the other hand, the compound is dispensed directly from 100% stock solutions, and sudden additions of solvent-altering components are in general stressful for protein crystals owing to osmotic effects (López-Jaramillo *et al.*, 2002 ▸), and possibly also owing to mechanical stress as a results of the high-frequency (200 Hz) pulses of solvent droplets. We therefore investigated how the positional precision of acoustic transfer could be exploited to ensure that soaking was not only rapid but also sufficiently gentle.

All of the dispensing patterns in Fig. 2 ▸ (Figs. 2 ▸ *c*–2 ▸ *h*) were investigated with JMJD2D crystals in order to assess which could provide the highest solvent concentration and thus the highest potential compound-soaking concentration, without significantly reducing the quality of X-ray diffraction. The patterns in Figs. 2 ▸(*c*) and 2 ▸(*d*) have a single target location, whereas those in Figs. 2 ▸(*e*)–2 ▸(*h*) have multiple target locations, with the total transfer volume spread across all locations. Each of the patterns was tested in duplicate with final solvent concentrations ranging from 10 to 60%(*v*/*v*) and soaking times of 1 h or overnight (96 soaking experiments). Crystal survival was measured by the collection of a full X-ray diffraction data set for each crystal, with the results from automatic data processing being used to gauge the outcome of different treatments. JMJD2D crystals mostly provide a binary readout, with crystals either providing a good-quality data set or displaying a complete loss of observable X-ray diffraction spots (and therefore no possible data-processing statistics), depending on the treatment, with very few crystals being of intermediate or poor quality.

**Figure 3:**
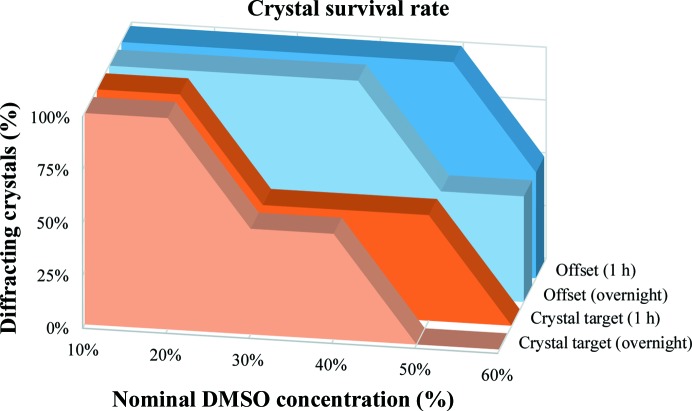
Offset targeting increases solvent tolerance and enables longer soaking times. Survival rate of JMJD2D crystals (percentage of diffracting crystals) from soaking in DMSO after acoustic transfer targeted at the crystal (orange) or targeted away from the crystal (blue). Crystals were soaked for 1 h (lighter colousr) or overnight (darker colours).

It was found that the crystals tolerate twice the solvent concentration when targeting away from the crystal with the offset approach (Fig. 2 ▸ *c*) compared with when the crystal was directly targeted (Fig. 2 ▸ *d*). Fig. 3 ▸ shows the survival rate, as a percentage of diffracting crystals, for 48 JMJD2D crystals that were soaked with the two methods at different final DMSO concentrations. Targeted crystals tolerated a 20%(*v*/*v*) final solvent concentration, whereas offset targeting provided a tolerance of 40%. All crystals have undergone an identical cryoprotection protocol (§[Sec sec2.5]2.5). Control crystals that were cryoprotected but did not receive DMSO treatment provided similar quality X-ray diffraction. Data-processing statistics for the crystals summarized in Fig. 3 ▸ (and control crystals) are presented in Supplementary Table S1. The different ring patterns of Echo dispensing, which were also investigated, produced intermediate crystal survival rates, with crystals tolerating up to a 30%(*v*/*v*) final DMSO concentration (data not shown).

### 3.3. Diffusion   

Typical crystal-soaking experiments usually involve preparing the compound of interest in a crystal-compatible solution, usually reservoir solution with an additional solvent such as DMSO, after which either the solution is transferred directly to the crystallization drop or the crystal is moved to the solution. This exposes the crystal to sudden changes from its native solution, which can damage the crystal through osmotic shock (López-Jaramillo *et al.*, 2002 ▸). Some crystals are far more tolerant to this form of treatment than others, but careful stepwise procedures can overcome this for those that are not, enabling higher solvent and compound concentrations to be introduced gradually to the crystal (Hassell *et al.*, 2007 ▸).

**Figure 4:**
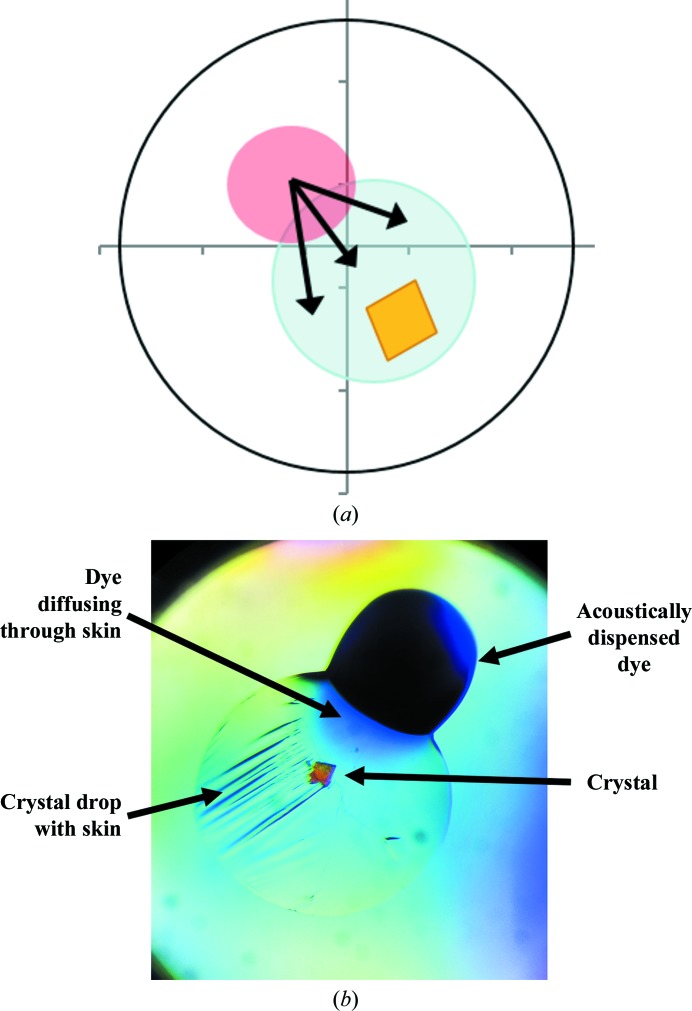
Compound diffusion after offset targeting through a droplet takes just several minutes. (*a*) Schematic illustration of diffusion (black arrows) from a crystallization droplet (blue circle) soaked with the offset targeting approach (the red circle represent the location of dispensed solvent). (*b*) Reduced diffusion of methylene blue dye through a crystallization drop that has a skin.

This provides the most likely explanation for why crystals tolerate the very high solvent concentrations generated by the acoustic offset targeting described here. Gradual diffusion of solvent/solute across the drop gives the crystal a significantly longer equilibration time (Fig. 4 ▸ *a*). A similar observation has been reported when compounds are delivered *via* a laser-generated aperture (Zander *et al.*, 2016 ▸). We observe that it takes 2–5 min for coloured compounds or dyes to diffuse across the drop and reach an equilibrium after acoustic transfer to the edge of a crystallization drop. In contrast, crystals that are plunged into a drop containing a new solvent, or are flooded by the addition of solvent, will experience equilibration within milliseconds or seconds at best, which is 2–5 orders of magnitude faster than gradual diffusion from offset targeting.

When a skin is present on the crystallization drop it acts as a membrane partition between the crystal drop and the transferred solution, but still allows gradual diffusion of solutes (Fig. 4 ▸ *b*). The process is however slower only by an order of magnitude, minutes to hours as judged by colour equilibration, depending on the age of the drop and the thickness of the skin. Disruption of the skin with a loop or microtool results in influx of the compound over a number of minutes as observed in the absence of a skin. Overall, a skin does not prevent acoustic crystal soaking; however, appropriate soaking times will need to be established.

The fast equilibration observed here stands in contrast to the long soaking times frequently mentioned anecdotally by experienced crystallographers as necessary for even high-affinity binders to show up in the crystal. However, in our experience, this is necessary to overcome low solubility (and sometimes to accommodate packing rearrangements), rather than to compensate for weak affinity.

Offset targeting is now the default protocol for the XChem platform for three reasons. Firstly, it allows very high solvent concentrations to be dispensed, and it is reasonable to assume that a correspondingly high compound concentration is important to ensure that the weakly binding fragments bind with sufficient occupancy to ensure detection in electron-density maps. Secondly, offset targeting is simple to perform, since a single click in *TeXRank* defines the target. Thirdly, the overall dispensing speed is considerably faster for a single target compared with the more complex patterns, which require many additional stage movements within the Echo (§[Sec sec3.4]3.4).

The same targets used for compound dispensing can also be used for adding cryoprotecting solutions to the drop prior to harvesting. Ethylene glycol is a convenient cryoprotectant since acoustic transfer can be performed directly from a 100% stock solution. The higher viscosity of glycerol requires a 50%(*v*/*v*) diluted stock solution for successful acoustic transfer, and thus requires larger volumes to be added to achieve the required cryoprotecting concentration. However, for routine fragment screening at Diamond, we, like others (Pellegrini *et al.*, 2011 ▸; Zander *et al.*, 2016 ▸), have observed that by matching the mounting loop to the crystal size and limiting excessive solvent surrounding the crystal, cryoprotection is often not required (§[Sec sec3.6]3.6).

### 3.4. Speed and throughput   

Acoustic dispensing is rapid for small nanolitre-scale volumes once the experiment has been designed and a transfer list constructed, as described in §§[Sec sec2.2]2.2 and [Sec sec2.4]2.4. The fixed rate of droplet ejection, 2.5 nl at 200 Hz, indicates a fluid-transfer rate of 500 nl s^−1^; however, for the large numbers of <100 nl transfers typical for fragment screening, the actual throughput is limited by stage movements rather than fluid transfer. For example, 1000 × 25 nl transfers take 7 min, but only 8.5 min is required for the same number of 100 nl transfers (62 and 196 nl s^−1^, respectively). Therefore, significant changes in transfer volumes have only a marginal impact on total transfer times at these scales, which correspond to the usual crystallization drop volumes typical for robotically prepared crystallization experiments (100–200 nl).

In practice, therefore, the transfer of 1000 unique compounds to 1000 unique crystallization-drop locations can be performed in under 10 min. This includes the time taken for stage movements within the Echo, and unsealing and resealing 4–5 crystallization plates full of crystals.

For the typical crystallization drop sizes cited above, the 1–2 min time frame for acoustic dispensing per plate is short enough that the plate seal can be completely removed during transfer without evaporation affecting the drops or the crystal integrity (results not shown). The internal chamber of the Echo is also somewhat humidified, as the sound waves are coupled from the transducer to the bottom of the source plate by flowing water, and presumably this helps to slow down evaporation.

For smaller drop volumes or volatile crystallization components, the experiment can be broken into batches by exposing smaller sections of the plate at a time; the only drawback is that the overall experiment takes more time. It has also been reported that the use of a plate lid containing small apertures that allow acoustic transfer but minimize disruption of the vapour environment around the drop significantly reduces evaporation and improves X-ray diffraction consistency (Zipper *et al.*, 2014 ▸).

### 3.5. Time and concentration   

We explored what amounts to the dynamic range of crystal soaking for detecting small-molecule binding by investigating how to modulate acoustic transfer soaking protocols for differently behaving compounds to ensure that they were detected. Three compounds were selected from different parts of the range, according to how strongly they had previously presented in the electron density.

Two molecules identified from fragment screening of JMJD2D (detailed report in preparation; models available from http://www.thesgc.org/fragment-screening) were selected and categorized as a *medium* binder and a *weak* binder, based on the signal from previously observed electron-density maps. These fragments did not display binding in biophysical screens, which can be expected given that they are already at the edge of detection for X-ray crystallography, especially for the weak binder. This correlates with the generally poor overlap observed for binding detection between different methods of ligand screening (Schiebel *et al.*, 2016 ▸). Also included was compound KDOAM16 (Bavetsias *et al.*, 2016 ▸; §[Sec sec3.1]3.1), which was designated a *strong* binder.

**Figure 5:**
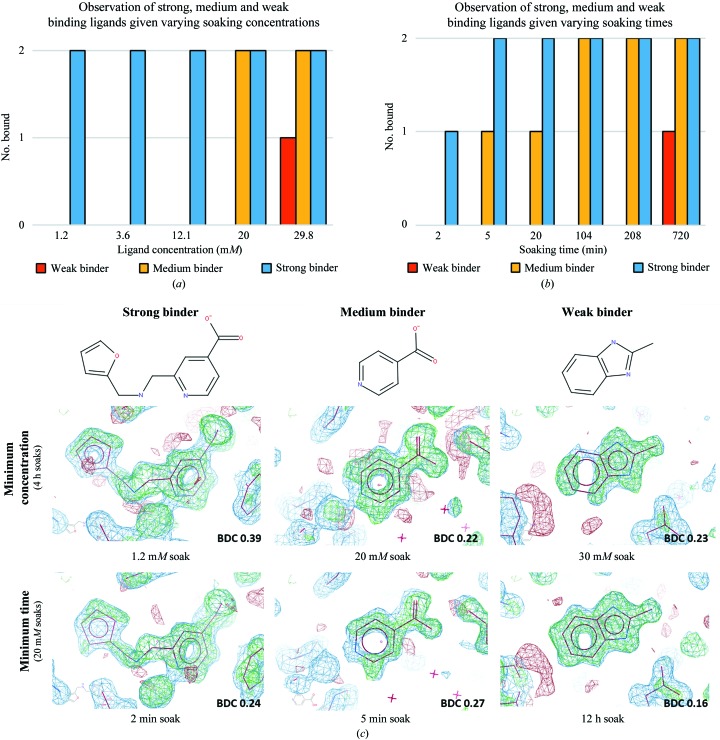
Variation of soaking times and concentration can be used to improve the visibility of the ligand. Plots showing the number of ligands detected (strong, medium and weak binding ligands) as a function of (*a*) concentration or (*b*) time after crystal soaking using acoustic transfer (from 100 m*M* stock). The concentration series in (*a*) were soaked for a fixed time of 4 h, while the time series in (*b*) were soaked at a fixed nominal concentration of 20 m*M*. Soaks were performed in duplicate for each condition, leading to 30 X-ray diffraction data sets for (*a*) and 36 data sets for (*b*). (*c*) Electron-density maps (*PanDDA* maps: event maps are shown in blue at 2σ, *Z*-maps are shown in green/red at ±3σ; 1.3–1.4 Å resolution) from the minimum experimental conditions (time or concentration series) required to detect ligand binding. The maps are generated prior to refinement of the ligand with the model. The ligand coordinates have been modelled into the electron-density maps for clarity. The* PanDDA* reported background density correction (BDC) values are shown (Pearce *et al.*, 2016 ▸).

Fig. 5 ▸(*a*) shows the detection threshold of the three molecules as a function of concentration. A compound was objectively considered detected if it passed the *Z*-map threshold in *PanDDA* with the default settings, as described in Pearce *et al.* (2016 ▸). The weak binder was only observed at 30 m*M* concentration, and then only for one out of the two duplicates, while the medium binder was not detected below 20 m*M*. The strong binder was detected for both duplicates at the minimum concentration tested of 1.2 m*M*. This corresponded to a single 2.5 nl acoustic droplet transferred to the crystallization drop, and shows that for tight-binding ligands the use of acoustic transfer is extremely effective, requiring only very limited amounts of compound. Two 2.5 nl transfers directly from the intact 100 m*M* stock (116 ng total compound) were sufficient to obtain two separate protein–ligand complex structures (Fig. 5 ▸ *c*). The results for the medium and weak binding ligands highlight the importance of compound concentration in order to detect weak binding ligands. Since the crystals have a ceiling on solvent tolerance, the next best way to increase compound concentration is to increase the concentration of the stock solution, although in practice compound solubility will dictate the effective concertation that can be achieved either in the stock solution or after addition to the crystallization drop.

The detection of binding for the three molecules from soaking for different lengths of time after acoustic transfer (soaking at a nominal 20 m*M* final concentration) is shown in Fig. 5 ▸(*b*). For the strong binder, only one of the duplicates was observed when the crystals were mounted 2 min after transfer. Care was taken when mounting to pick the crystal directly out of the drop without excessive mixing of solvent within the drop, in order to isolate the effect of compound diffusion across the drop; nevertheless, some mixing will inevitably have occurred. For the medium binder, 2 min was insufficient for the ligand to be detected, while at 5 min one of the duplicates was detected (Fig. 5 ▸ *c*). The weak binder was only detected after an overnight soak at 20 m*M*. Supplementary Fig. S2 shows, similarly to Fig. 5 ▸(*c*), the electron-density maps (*PanDDA* *Z*-maps) for soaks just below the detection threshold for time or soaking concentration.

### 3.6. The importance of control experiments   

As discussed above, the dynamics of adding small amounts of 100% stock solutions directly to a crystallization drop for crystal soaking are different compared with manually transferring a crystal to a new drop or flooding it with a pre-prepared solution. From many subsequent experiments (not reported here), we conclude that previous knowledge of the solvent tolerance of a crystal system tends to be unrelated to that observable by acoustic dispensing with offset targeting, which typically permits higher solvent concentrations.

The implication is that thorough advance control experiments are essential for establishing a maximally effective offset targeting protocol. A standard solvent-tolerance screen has been implemented that involves testing X-ray diffraction from crystals soaked at 5–40%(*v*/*v*) final DMSO concentration at three time points (1 h, 3 h and overnight) in duplicate, and including untreated control crystals (30–36 crystals in total), similar to the characterization described in §[Sec sec3.2]3.2 for JMJD2D. In this way, the optimal crystal-soaking parameters can be determined in a systematic manner for each new crystal system entering into a compound-soaking campaign. While some crystal systems display a sudden transition from well diffracting to zero diffraction at higher solvent concentrations, like JMJD2D, others display a more general degradation of X-ray diffraction quality with increased solvent concentrations and soaking times. The crystallographer will select an optimal condition (based on data-processing statistics, typically resolution, and signs of crystal pathologies from the X-ray diffraction patterns) that yields quality X-ray diffraction, similar to the untreated control crystals, while still providing the maximal solvent concentrations and soaking times. Finally, the real-life validity of the parameters are confirmed by performing an initial set of soaking experiments (10–100) with small molecules/fragments, to ensure that diffraction is indeed consistently retained, before launching into a full fragment screen.

**Figure 6:**
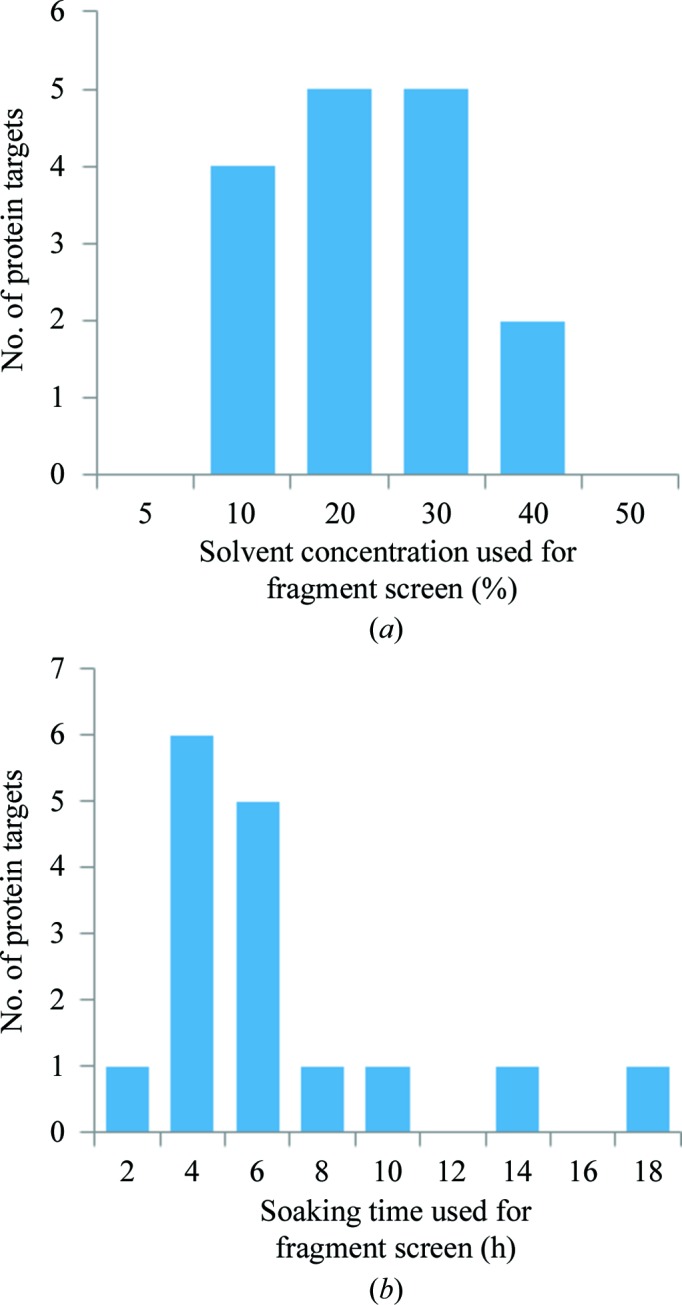
Individual adjustment of solvent concentration and soaking time is essential for successful soaking experiments. The working soaking conditions selected and used for 16 recent protein targets which were investigated as part of the XChem user program at the Diamond Light Source. The graphs show (*a*) the nominal final DMSO concentration used for fragment screening and (*b*) the soaking time selected for each project. A total of over 18 000 crystals were soaked in the course of these experiments.

From over 18 000 crystal soaks across 16 recent protein targets, the optimally selected DMSO solvent concentration is found to range between 10 and 40% (23% on average; of course, the actual concentrations are likely to be underestimated by as much as half, as discussed in §[Sec sec2.2]2.2), with soaking times of 4–6 h (Fig. 6 ▸). Additionally, 11 of the 16 targets have not required further cryoprotection. The broader outcomes from many X-ray fragment-screening campaigns, across multiple varied protein families, will be reported in more detail in a separate publication.

## 4. Conclusions   

We have developed a method for the soaking of protein crystals that is both gentle and rapid. By using the precision of acoustic dispensing to target the transfer of solvent and compounds away from sensitive protein crystals, an increase in solvent tolerance and X-ray diffraction reproducibility can be achieved, even at a rate of 100 crystals per minute. The use of control experiments to empirically determine the optimal experimental conditions for each crystal system has enabled fragment screening on multiple diverse protein targets in the XChem facility at Diamond. We typically observe fragment hit rates of between 1 and 10%, which is in good agreement with what other groups have observed (Bauman *et al.*, 2013 ▸; Schiebel *et al.*, 2016 ▸; Spurlino, 2011 ▸); hence, we conclude that the described protocol works efficiently. While the method may not be ideal for all crystal systems, its great advantage is that it is generic, since it can be applied to any crystal system with minimal preparatory work. The solvent-tolerance screen provides a means to detect problems in the soaking protocol in a systematic manner and avoids time being lost on soaking, collecting and analysing fragment-soaked crystals with little chance of success. A fast and efficient soaking protocol is instrumental for the XChem user program at Diamond Light Source, and the more than 30 000 crystals that have been soaked and data collected from them and the 800 fragment hits that have been seen so far are testament to its robustness.

## 5. Data availability   

The X-ray structures presented here are part of a larger fragment screen, with data available from the Zenodo data repository, https://doi.org/10.5281/zenodo.345833.

## Supplementary Material

Supporting Information.. DOI: 10.1107/S205979831700331X/ba5268sup1.pdf

Fragment screen data. URL: https://doi.org/10.5281/zenodo.345833
