# Supplementary material for: Gentle, fast and effective crystal soaking by acoustic dispensing
Source: Acta Crystallogr D Struct Biol. 2017 Mar 6;73(Pt 3):246–55. doi: 10.1107/S205979831700331X (PMC5349437; doi:10.1107/S205979831700331X)
Supplement: Supplementary file 1 [file d-73-00246-sup1.pdf]

STRUCTURAL
BIOLOGY

Volume 73 (2017)

Supporting information for article:

Gentle, fast and effective crystal soaking by acoustic dispensing

Patrick M. Collins, Jia Tsing Ng, Romain Talon, Karolina Nekrosiute, Tobias Krojer, Alice Douangamath, Jose Brandao-Neto, Nathan Wright, Nicholas M. Pearce and Frank von Delft

S1. TeXRank output csv file format

The output format for TeXRank is a csv file with 4 columns. For a SWISSCI 3 drop plate, the file contains all 288 wells (288 lines in the file) (no header), with those selected for targeting containing the *x y* coordinates. Column 1: Well Number, Column 2: *x* offset value, Column 3: *y* offset value, Column 4: a score (default = 6)

For example, the first 5 lines of a TeXRank output file where 3 drops have been targeted, and 2 wells are not targeted.

```
A01a,-61,763,6
A01c,,,
A01d,57,893,6
B01a,-141,324,6
B01c,,,
```

S2. Echo region definition input file format

The region definition input file for the Labcyte Plate Reformat software is a csv file with 6 columns, and includes a header row. Column 1: Plate name, Column 2: Source well, Column 3: Destination well, Column 4: Transfer Volume, Column 5: Destination Well X offset, Column 6: Destination Well Y Offset.

For example, the first 6 lines of an Echo region definition csv file:

```
PlateBatch,Source well,Destination well,Transfer Volume,Destination Well X
Offset,Destination Well Y Offset
441332000038_B1,A10,C1,0,672,-1226
441332000038_B1,A10,D1,0,429,-247
441332000038_B1,A10,D2,28,-460,-747
441332000038_B1,A10,E1,28,958,306
441332000038_B1,A10,F1,55,1026,-763
```

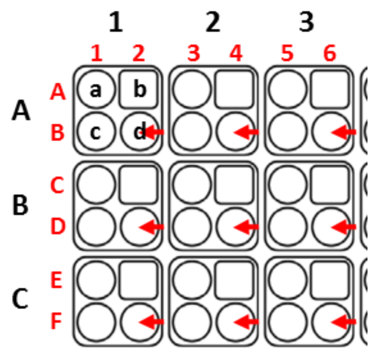

Figure S1 Top left corner of a SWISSCI 3 lens crystallisation plate. The normal definition of the plate has 96 locations, with four subwells per location (black text). We define the plate in a 384-well format (red text) and apply an offset correction to the even-numbered columns (red arrows), which are not positioned centrally between adjacent subwells.

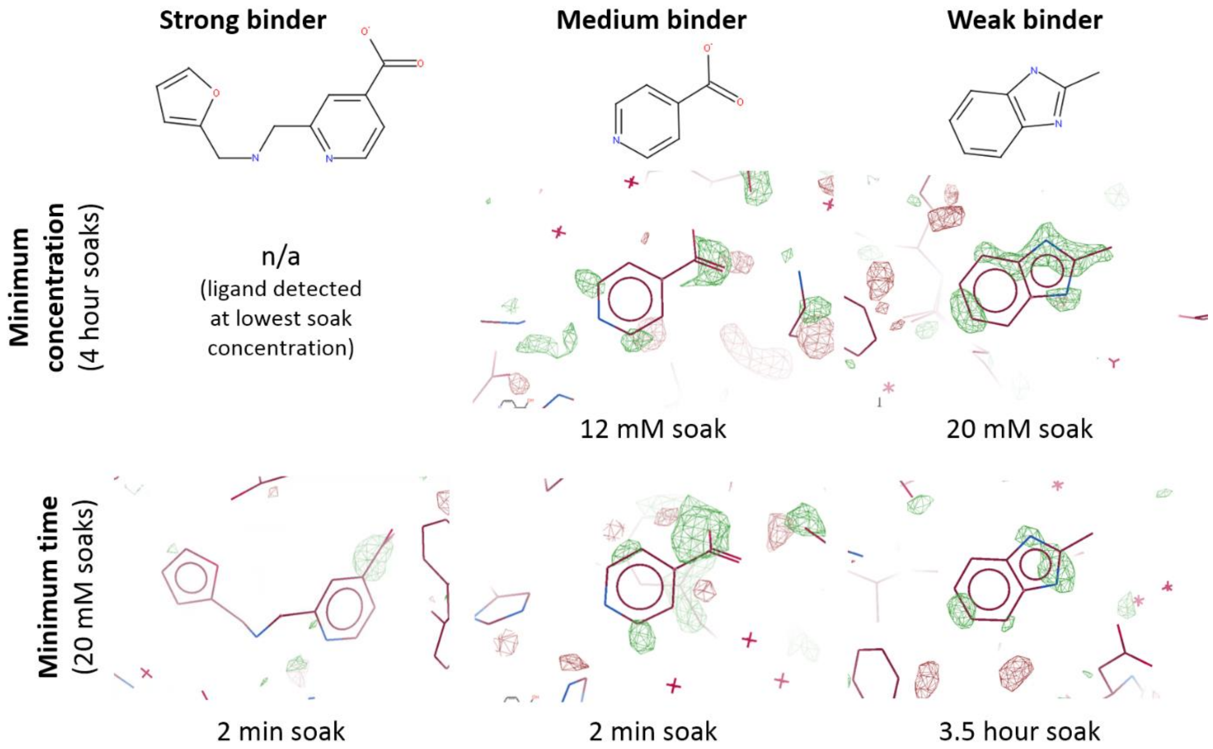

Figure S2 Soaking conditions where ligands were not detected. Electron density maps (PanDDA maps (Pearce *et al.*, 2016): Z-maps: green/red, ±3, 1.3–1.5 Å resolution) from the experimental conditions (time or concentration series) below the threshold required to detect ligand binding. The maps are viewed from the same position as Figure 5c in the main text. The ligand coordinates are included for references in the same positions as modelled in Figure 5c. Note that unlike Figure 5c, this figure only shows PanDDA Z-maps, and not event maps, since event maps are only generated when a binding event is detected.

Table S1 Summary of X-ray diffraction data processing statistics for JMJD2D crystals soaked at various DMSO concentrations (final DMSO % (v/v)), after acoustic transfer of solvent either targeted away from the crystal (offset), or at the crystal (target).

Crystals were soaked for either 1 hour, or overnight. Data processing statistics are from *xia2* (the *Aimless* step), and refinement statistics (Rcryst and Rfree) are from the dimple pipeline (Refmac restrained refinement).

Crystal Name	Target type	DMSO (%)	Soak Time	Resolution (High)	Resolution (Low)	Rmerge (Overall)	Rmerge (Low)	Rmerge (High)	Isig (Overall)	Isig (Low)	Isig (High)	Completeness (Overall)	Rcryst	Rfree
JMJD2DA-MB-217	control	0%	na	1.28	29.49	0.057	0.026	0.704	23.6	83.8	1.9	99.1	0.183	0.196
JMJD2DA-MB-307	control	0%	na	1.21	29.53	0.055	0.030	0.701	27.8	79.2	3.1	80.0	0.179	0.196
JMJD2DA-MB-x934	offset	10%	1 hour	1.58	28.14	0.081	0.024	0.875	23.9	82.9	3.0	99.8	0.187	0.216
JMJD2DA-MB-x936	offset	10%	1 hour	nc	nc	nc	nc	nc	nc	nc	nc	nc	nc	nc
JMJD2DA-MB-x935	offset	20%	1 hour	2.25	65.91	0.549	0.115	0.530	3.8	14.7	3.7	94.3	0.309	0.379
JMJD2DA-MB-x937	offset	20%	1 hour	1.47	52.75	0.108	0.029	2.262	15.7	60.1	1.4	99.8	0.195	0.224
JMJD2DA-MB-x927	offset	30%	1 hour	1.54	29.59	0.074	0.024	0.800	25.0	86.3	3.5	99.8	0.180	0.210
JMJD2DA-MB-x927	offset	30%	1 hour	1.54	29.59	0.074	0.024	0.800	25.0	86.3	3.5	99.8	0.180	0.210
JMJD2DA-MB-x929	offset	40%	1 hour	1.65	29.51	0.094	0.026	0.871	22.5	86.3	3.4	99.8	0.178	0.210
JMJD2DA-MB-x929	offset	40%	1 hour	1.65	29.51	0.094	0.026	0.871	22.5	86.3	3.4	99.8	0.178	0.210
JMJD2DA-MB-x930	offset	50%	1 hour	1.59	29.59	0.092	0.032	0.866	20.2	66.3	3.0	99.7	0.179	0.212
JMJD2DA-MB-x931	offset	50%	1 hour	1.54	50.80	0.153	0.030	2.669	14.9	74.3	1.2	99.8	0.197	0.230
JMJD2DA-MB-x932	offset	60%	1 hour	nd	nd	nd	nd	nd	nd	nd	nd	nd	nd	nd
JMJD2DA-MB-x933	offset	60%	1 hour	2.13	52.70	0.465	0.065	2.461	7.3	37.1	1.3	99.9	0.215	0.269
JMJD2DA-MB-x914	offset	10%	overnight	1.39	29.55	0.062	0.022	0.792	28.0	103.5	3.2	99.5	0.181	0.205
JMJD2DA-MB-x915	offset	10%	overnight	1.39	50.83	0.112	0.036	1.894	15.3	60.8	1.4	100.0	0.192	0.217
JMJD2DA-MB-x916	offset	20%	overnight	nc	nc	nc	nc	nc	nc	nc	nc	nc	nc	nc
JMJD2DA-MB-x917	offset	20%	overnight	1.25	72.46	0.103	0.056	1.537	12.8	42.9	1.3	99.9	0.192	0.214
JMJD2DA-MB-x918	offset	30%	overnight	1.66	29.43	0.077	0.022	0.832	26.6	92.4	3.3	99.9	0.178	0.209
JMJD2DA-MB-x920	offset	30%	overnight	1.60	52.32	0.171	0.035	2.139	12.7	58.1	1.3	99.9	0.190	0.221
JMJD2DA-MB-x919	offset	40%	overnight	1.25	52.31	0.129	0.077	1.603	10.5	32.0	1.2	100.0	0.193	0.215
JMJD2DA-MB-x921	offset	40%	overnight	1.72	64.87	0.229	0.058	2.481	9.4	34.5	1.2	100.0	0.205	0.245
JMJD2DA-MB-x922	offset	50%	overnight	nd	nd	nd	nd	nd	nd	nd	nd	nd	nd	nd
JMJD2DA-MB-x924	offset	50%	overnight	1.79	29.52	0.110	0.035	0.789	19.5	63.2	3.6	99.9	0.202	0.236
JMJD2DA-MB-x923	offset	60%	overnight	nd	nd	nd	nd	nd	nd	nd	nd	nd	nd	nd
JMJD2DA-MB-x925	offset	60%	overnight	1.61	52.45	0.164	0.034	2.252	13.5	61.3	1.4	99.9	0.190	0.219
JMJD2DA-MB-x891	target	10%	1 hour	1.36	72.69	0.115	0.040	1.748	14.3	52.6	1.5	99.3	0.190	0.214
JMJD2DA-MB-x894	target	10%	1 hour	1.38	72.62	0.145	0.088	1.615	9.6	25.9	1.5	100.0	0.206	0.241
JMJD2DA-MB-x890	target	20%	1 hour	1.31	29.86	0.080	0.050	0.777	18.7	51.4	2.8	100.0	0.183	0.205
JMJD2DA-MB-x899	target	20%	1 hour	2.18	151.65	0.889	0.241	2.908	5.9	11.0	2.7	100.0	0.219	0.287
JMJD2DA-MB-x895	target	30%	1 hour	1.60	29.79	0.102	0.029	0.856	21.0	75.2	3.5	99.9	0.179	0.209
JMJD2DA-MB-x897	target	30%	1 hour	nd	nd	nd	nd	nd	nd	nd	nd	nd	nd	nd
JMJD2DA-MB-x892	target	40%	1 hour	1.24	52.36	0.136	0.092	1.663	9.9	28.1	1.2	100.0	0.192	0.214
JMJD2DA-MB-x893	target	40%	1 hour	nd	nd	nd	nd	nd	nd	nd	nd	nd	nd	nd
JMJD2DA-MB-x900	target	50%	1 hour	2.63	29.69	0.092	0.043	0.166	31.6	48.0	18.3	99.1	0.208	0.282
JMJD2DA-MB-x901	target	50%	1 hour	nd	nd	nd	nd	nd	nd	nd	nd	nd	nd	nd
JMJD2DA-MB-x896	target	60%	1 hour	nd	nd	nd	nd	nd	nd	nd	nd	nd	nd	nd
JMJD2DA-MB-x898	target	60%	1 hour	nd	nd	nd	nd	nd	nd	nd	nd	nd	nd	nd
JMJD2DA-MB-x903	target	10%	overnight	1.60	29.62	0.100	0.041	0.784	18.9	55.2	3.6	99.9	0.179	0.212
JMJD2DA-MB-x909	target	10%	overnight	1.53	29.49	0.087	0.038	0.861	20.5	61.5	3.2	99.8	0.182	0.209
JMJD2DA-MB-x904	target	20%	overnight	1.41	52.56	0.078	0.025	1.603	22.0	88.1	1.7	99.9	0.189	0.215
JMJD2DA-MB-x905	target	20%	overnight	1.50	29.64	0.085	0.037	0.814	20.7	62.0	3.3	99.9	0.180	0.208
JMJD2DA-MB-x902	target	30%	overnight	1.55	29.61	0.081	0.030	0.877	22.7	74.3	3.1	99.8	0.182	0.215
JMJD2DA-MB-x913	target	30%	overnight	nd	nd	nd	nd	nd	nd	nd	nd	nd	nd	nd
JMJD2DA-MB-x908	target	40%	overnight	nd	nd	nd	nd	nd	nd	nd	nd	nd	nd	nd
JMJD2DA-MB-x910	target	40%	overnight	1.29	50.75	0.108	0.066	1.833	12.5	38.5	1.2	100.0	0.192	0.213
JMJD2DA-MB-x906	target	50%	overnight	nd	nd	nd	nd	nd	nd	nd	nd	nd	nd	nd
JMJD2DA-MB-x907	target	50%	overnight	nd	nd	nd	nd	nd	nd	nd	nd	nd	nd	nd

JMJD2DA-MB-x911	target	60%	overnight	nd	nd	nd	nd	nd	nd	nd	nd	nd	nd	nd
JMJD2DA-MB-x912	target	60%	overnight	nd	nd	nd	nd	nd	nd	nd	nd	nd	nd	nd

nd = no diffraction (there were no observable X-ray diffraction spots from the crystal, or spots were of such poor quality that images could not be processed to give statistics).

nc = not collected (X-ray diffraction data was not collected due to an external error)
